# X-ray-based virtual slicing of TB-infected lungs

**DOI:** 10.1038/s41598-019-55986-y

**Published:** 2019-12-18

**Authors:** Ana Ortega-Gil, Juan José Vaquero, Mario Gonzalez-Arjona, Joaquín Rullas, Arrate Muñoz-Barrutia

**Affiliations:** 1 Departamento de Bioingeniería e Ingeniería Aeroespacial, Universidad Carlos III de Madrid, Leganés, Spain; 2 Instituto de Investigación Sanitaria Gregorio Marañón (IiSGM), Madrid, Spain; 3 Diseases of the Developing World, Infectious Diseases-Centre for Excellence in Drug Discovery (ID CEDD), GlaxoSmithKline, Madrid, Spain

**Keywords:** Medical research, Preclinical research

## Abstract

Hollow organs such as the lungs pose a considerable challenge for *post-mortem* imaging in preclinical research owing to their extremely low contrast and high structural complexity. The aim of our study was to enhance the contrast of tuberculosis lesions for their stratification by 3D x-ray–based virtual slicing. Organ samples were taken from five control and five tuberculosis-infected mice. Micro-Computed Tomography (CT) scans of the subjects were acquired *in vivo* (without contrast agent) and *post-mortem* (with contrast agent). The proposed contrast-enhancing technique consists of x-ray contrast agent uptake (silver nitrate and iodine) by immersion. To create the histology ground-truth, the CT scan of the paraffin block guided the sectioning towards specific planes of interest. The digitalized histological slides reveal the presence, extent, and appearance of the contrast agents in lung structures and organized aggregates of immune cells. These findings correlate with the contrast-enhanced micro-CT slice. The abnormal densities in the lungs due to tuberculosis disease are concentrated in the right tail of the lung intensity histograms. The increase in the width of the right tail (~376%) indicates a contrast enhancement of the details of the abnormal densities. *Postmortem* contrast agents enhance the x-ray attenuation in tuberculosis lesions to allow 3D visualization by polychromatic x-ray CT, providing an advantageous tool for virtual slicing of whole lungs. The proposed contrast-enhancing technique combined with computational methods and the diverse micro-CT modalities will open the doors to the stratification of lesion types associated with infectious diseases.

## Introduction

Cutting-edge translational research on preclinical models of infectious lung diseases rely on computed tomography (CT) images for the assessment of infection burden and in particular, for the evaluation of new effective antibiotic combinations for tuberculosis (TB) treatment^1,2^.

The stratification of abnormal x-ray densities associated with tuberculosis is highly challenging in preclinical research owing to low lung contrast and the ground glass appearance of lesions. Contrast agents enhance the x-ray attenuation of soft-tissue and could allow 3D visualization of lesion composition when using a polychromatic x-ray CT scan^3–5^. The hypothesis driving this work was that the enhancement of complex lung structures by conventional contrast agents would allow the stratification of tuberculosis lesions by *post mortem* polychromatic x-ray imaging.

The use of contrast agents with a high atomic number (most commonly iodine or barium) for micro-CT imaging is extensively reported in the literature both for *in vivo* and *postmortem* studies^3,6,7^. Small iodinated agents are commonly injected into blood vessels to enable visualization of the vascular system and organ perfusion^8^ or for the estimation of the volumetric air, blood, and tissue fractions in the rodent lung when imaging with dual-energy scans^9^. Barium contrast medium is typically administered orally or with an enema to investigate the anatomy of the gastrointestinal tract^8^. For lung lesions, the number of options to perform longitudinal follow-up of the disease decreases notably. In the case of lung cancer models, iopamidol lipid, iodinated lipid, and inorganic nanoparticulate agents enable differentiation between the intensities of tumours, vascular structures, and thoracic air space and thus, identification of tumour margins and accurate quantification^10^. *Postmortem* studies also benefit from the use of contrast agents, as micro-CT imaging of excised organs (e.g., heart, lung, brain)^11,12^ and even whole animals (e.g., chick embryos)^11^ allow for 3D non-destructive virtual histology examination. This circumvents the challenges inherent to performing 3D histological reconstruction of the whole organ from serial sections, which is a difficult and laborious procedure.

**Figure 1:**
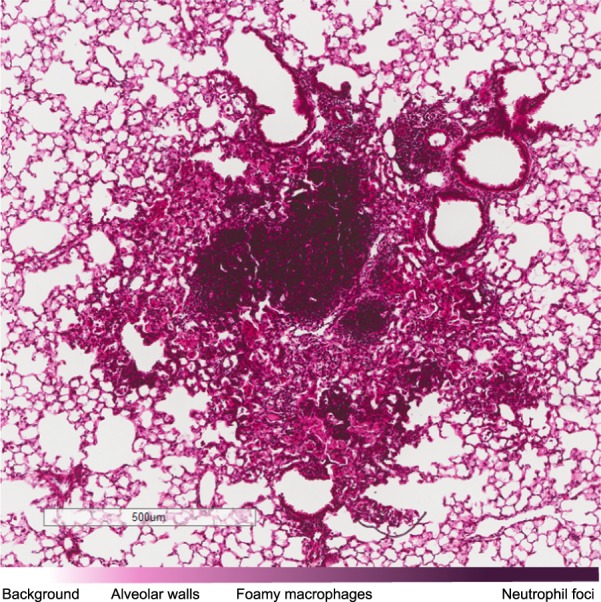
High-magnification histological slide of a tuberculous lesion, eight weeks after the infection in a murine model of the disease. The image shows the rim of foamy macrophages and a neutrophil focus. The colour scale helps the identification of the cells based on the haematoxylin-eosin stain, which enhance the nuclei and adds contrast for the cytoplasm and extracellular matrix.

Common criteria to characterize lesions histologically rely on organization: ‘organized’ lesions are bounded by a peripheral cuff of lymphocytes^13–15^. Several models accurately reflect aspects of the progression of disease in macaques, marmosets^16–23^, zebrafishes^24,25^, and mice^13–15^. In this work, we chose a resistant murine model whose host-pathogen dynamics present in non-necrotic lesion and predominantly intracellular bacilli. The lesions present in our murine model are ‘unorganized’ and typically small. The cellular composition consists of two layers: a peripheral rim of foamy macrophages and a central cup of neutrophil focus^26,27^ (Fig. 1).

The aim of our study was to enhance tuberculosis lesions for 3D x-ray–based virtual slicing. The proposed contrast-enhancing technique consists of high-atomic number contrast agent uptake by immersion. Aware of the limitations and resolution differences with classical histology, we will use the term x-ray based virtual slicing to refer to the 3D section visualization with enough resolution to identify lung structures in whole organs already prepared for classical histological analysis. The resolution of the x-ray based virtual slicing is determined by the imaging platform (44um, in our case). To illustrate that the enhancement can be achieved with multiple contrast agents, we present two alternatives (silver nitrate and iodine) targeting the diagnostic x-ray energy range (10 keV, 150 keV)^9,28–30^.The contrast-enhanced organs are embedded in ethanol and then moved to paraffin, which enables longer storage of the biological phantom at room temperature. The histological analysis of our samples reveals the distribution of the contrast agent in the lesion subtypes associated with the tuberculosis model under study and the nature of their abnormal x-ray density.

## Materials and Methods

**Figure 2:**
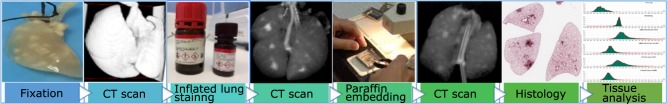
Flow chart for the contrast-enhancing technique: Inflated fixed lungs are immersed in the contrast agent solution. After 14 hours, they are stored in ethanol, and a micro-CT scan is performed. The organ is then embedded in paraffin and a second micro-CT scan is performed. Finally, after preparation with haematoxylin and haematoxylin-eosin, slices are digitized using optical microscopy. Micro-CT scans and histopathological images are ready for visual inspection and quantitative analysis.

The sequential steps of the contrast-enhancing technique are presented in Fig. 2. The technique was based on excised whole organs from procedures ethically reviewed and carried out in accordance with European Directive 2010/63/EEC and the GSK Policy on the Care, Welfare and Treatment of Animals. This experimental protocol entitled “Búsqueda de nuevos tratamientos frente a la tuberculosis”, with project number PROEX 63/14, was approved by the Consejería de Medio Ambiente de la Comunidad de Madrid. The mouse organs were excised from ten females of the C57BL/6 J strain. Five were inoculated with 100 CFUs of the virulent *tuberculosis* H37Rv strain at the age of 8−12 weeks. The Tuberculosis strain was originally disassociated by Steenken *et al*.^31^, and the infection protocol was optimized by our team. All animals were sacrificed eight weeks after the intratracheal insult. At that time, the infected mice had reached the chronic phase of the disease. Before the lungs were extracted, 1.5 ml of 10% formalin were instilled through the trachea.

### Inflated lung contrast-enhancement

The mouse organs were dehydrated by graded ethanol concentration fixation (GECF), a prior biochemical conditioning step described by de Silva *et al*.^6^. GECF begins with 50% ethanol for one hour and proceeds with ethanol solutions at increasing concentrations (70, 80, 90, 96 and 100%) for one hour each.

After fixation, eight of the ten samples were immersed in the contrast agent solution for 14 hours. Two dissected healthy mouse lungs and two dissected TB-infected mouse lungs were immersed in silver nitrate (Sigma-Aldrich Co.) in 3% w/v ethanol solution (saturated). Two dissected healthy mouse lungs and two dissected TB-infected mouse lungs were immersed in iodine (Sigma-Aldrich Co.) in 3% w/v ethanol solution. The two remaining samples (one dissected healthy mouse lung and one dissected tuberculosis-infected mouse lung) were also prepared for histology without any contrast agent and served as controls.

To prevent abrupt shrinkage of the fixed sample and ensure homogeneous uptake throughout the subsequent immersions (GECF and contrast agent), the organs were kept in suspension at a constant pressure of 20 cmH_2_O by connecting a computerized pneumatic circuit to the tracheal catheter. The pressure system keeps the lungs inflated in suspension to guarantee a homogeneous uptake, due to the subtle fluid stream. It also serves to prevent the lung tissue from collapsing, even in the case of perforation. The system design can be found in the Supplementary Appendix A (Supplementary Fig. 1).

Samples were stored in a 2 ml plastic container filled with 70% ethanol to prevent further dehydration of the tissue. All the steps describing the contrast-enhancing protocol are summarized in the Supplementary Appendix B.

The whole organs were immersed in paraffin blocks to prepare for histological sectioning.

### Micro-CT whole lung and histological sections imaging

A standard micro-CT subsystem of a SuperArgus scanner (Sedecal Molecular Imaging, Madrid) was used for both *in vivo* and excised lung scans. The selected acquisition protocol was the manufacturer’s protocol for soft-tissue imaging tasks, which follows the “as low as reasonably achievable” principle stated in the Code of Federal Regulations (10 CFR 20.1003). Acquisition parameters are detailed in Table 1. Datasets were reconstructed using the filtered back-projection algorithm^32^.

**Table 1:** Micro CT acquisition protocols and parameters for each imagining task.

Imaging task	*In vivo*	Excised lungs
Scan type	Step and shoot	
Tube voltage	68 kVp	
Tube current	420 µA	
Filtration	0.2 mm Cu + 1.8 mm Al intrinsic	
Exposure time	50 ms	
Circular projection	514 over 360°	
Averaged projections per angular step	4	8
Magnification	3.3	
Pixel size (pixel binning)	0.29 mm (4 × 4)	0.14 mm (2 × 2)
Data volume (per frame)	2.17 GB (1.44 MB)	4.27 GB (2.88 MB)
Total acquisition time	8 minutes	16 minutes
Voxel size	88 × 88 × 88 µm	44 × 44 × 44 µm

The scanner was optimized for soft-tissue imaging in both cases in terms of x-ray filtration and detection sensitivity.

Before euthanizing the subjects, the ten mice were screened *in vivo* by micro-CT. These *in vivo* scans were acquired to confirm the infective stage and lesion location. The excised lungs were screened *post-mortem* two times: once the lungs uptook the contrast agent and were embedded in ethanol and once when embedded in paraffin (Fig. 2).

The axial 44-µm–thick CT slices of the paraffin blocks were acquired parallel to the microtome slicing plane, thus resulting in the aligned virtual volumes used to define the histology planes. Namely, planes of interest containing relevant tuberculosis lesions were selected on the micro-CT volume and their depth in micrometres from the paraffin surface was estimated (Supplementary Fig. 2). Three histological sections were processed per mouse: the first, with no further staining other than the contrast agent; the second, with haematoxylin to enhance the nuclei; and the third, with haematoxylin-eosin, which adds the contrast for the cytoplasm and extracellular matrix. In total, 30 slides were digitalized using the Aperio CS2 image capture device (Leica Biosystems Nussloch GmbH, De) at 40x magnification and 0.251 µm × 0.251 µm pixel size.

### Tissue analysis in micro-CT scans

First, the volume preservation of the processed samples was assessed. The morphometric parameters applied for the assessment of sample preservation on the micro-CT scans were the total volume of the healthy lung and the volume of the diseased tissues in the infected samples. The tissues of interest were identified in the micro-CT slices based on the difference in their grey levels, enhanced by the contrast agent.

Lung volumes are quantified using thresholds computed from the histograms of lung voxels in Hounsfield units (HU), as described in Chen, R *et al*.^33^. In brief, intensity windows can be depicted to individually capture lung tissue or apparent disease from the micro-CT scans. The thresholds delimiting each window are established to divide the HU histogram of the virtual volumes into the three parts above mentioned (healthy lung tissue, rim of foamy macrophages and neutrophil foci). Sample volumes were calculated by summing the total volume of the voxels belonging to each window. For the volumetric estimations, an expert radiologist selected the thresholds defining healthy tissue and disease associated tissue by visual inspection of the *in vivo* and the ethanol-embedded micro-CT scans. The thresholds for the paraffin-embedded micro-CT scans were selected using the histopathological labelling information by registering the micro-CT slices with histological slides and statistically modelling the HU distribution, as described in our previous work in Ortega-Gil, A *et al*.^34^. This methodology translates histopathological segmentation to micro-CT segmentation and provides a satisfactory estimation of the granuloma cellular structure. (Supplementary Table 1).

In the case of the contrast agents used for x-ray imaging, we studied the effect on the attenuation factor of the tissues. For this quantitative evaluation of contrast enhancement in lung tissue, we provide the edge-based contrast criterion (ECC) as a local index.

The ECC is defined as the average of the pixel contrast values^35,36^ and is computed as follows:

1 $$ECC=\frac{1}{UV}\mathop{\sum }\limits_{i=1}^{U}\mathop{\sum }\limits_{j=1}^{V}\frac{|I(i,j)-\underline{E}(i,j)|}{|I(i,j)+\underline{E}(i,j)|}$$

where U and V are the horizontal and vertical size of the image in pixels and I(i, j) is the grey level value at pixel (i, j). $\underline{E}$ is the mean edge grey level computed within a local N = 3 × 3 square window as given by:

2 $$\underline{E}(i,j)=\frac{{\sum }_{(k,l)\in N(k,l)}I(k,l)G(k,l)}{{\sum }_{(k,l)\in N(k,l)}G(k,l)}$$

where G is the Sobel gradient magnitude.

### Tissue analysis in histology

Histology is the gold standard for morphological tissue assessment. The tissue sections were analysed and labelled by three independent pathologists to evaluate the sample preservation after the preparation procedure in terms of obstructed/unobstructed airways and depiction of the lung cell types. Histology revealed the presence, extent, and appearance of the contrast agents in the tuberculosis lesions as indicators of the contrast enhancement in the micro-CT scans.

## Results

**Figure 3:**
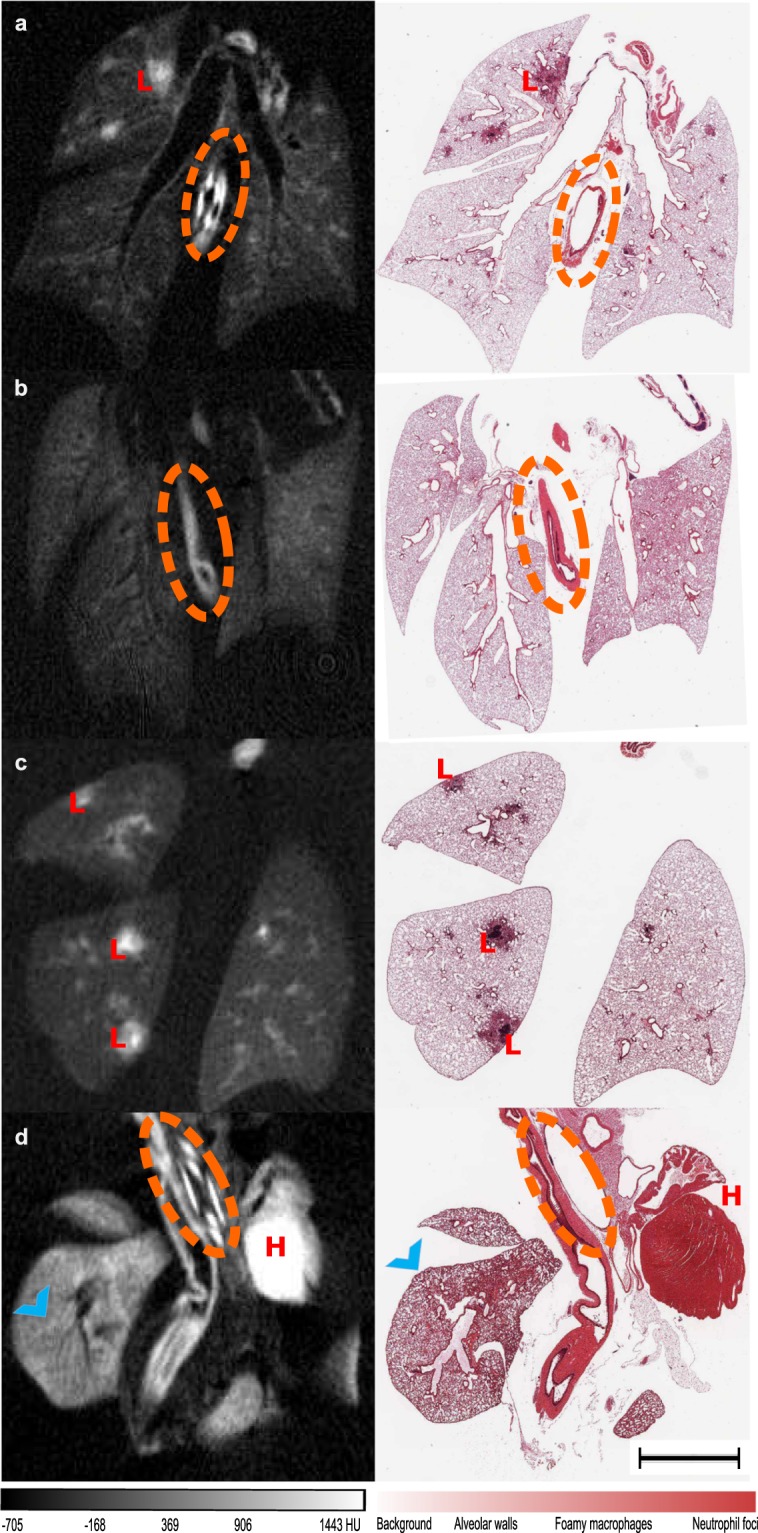
Micro-CT slices and their corresponding histology slide. Lung with silver nitrate uptake: (**a**) Infected; (**b**) Healthy. Lung with iodine uptake: (**c**) Infected; (**d**) Healthy. The airways are unobstructed (dashed orange circles), and the structure of the parenchyma has been preserved. Lesions (red L letter) are visible, with higher contrast uptake (higher intensities) at the central foci. High contrast agent precipitation is also present on the heart tissue (H red letter).Tissue preservation in (**d**) is reduced owing to complications during instillation at the isolation and fixation step, presenting collapsed alveolar walls (blue arrow head). The scale bar is common for all the thumbnails and measures 2 mm.

The micro-CT scans of the samples embedded in paraffin facilitate the selection of planes-of-interest for histological staining. Axial slices of the 3D micro-CT scans of the paraffin blocks and their corresponding histological slides are shown in Fig. 3 both for silver nitrate enhanced lungs (Fig. 3a infected; Fig. 3b healthy control) and iodine enhanced lungs (Fig. 3c infected; Fig. 3d healthy control). The contrast of the lungs with contrast agent is homogeneous as observed in the histological slides (Fig. 3, right panel). The dehydration step increases cell membrane permeation, and the contrast-agent molecules diffuses more easily into the tissue^6^.

The air spaces are homogeneously inflated, the septal walls are thin, and the corner vessels are open in the histological sections shown in Fig. 3a–c, suggesting successful perfusion fixation. In contrast, the section of the thoracic pluck shown in Fig. 3d presents areas of alveolar wall collapse (blue arrow head) and high contrast agent precipitation on the heart tissue (H red letter). This indicates that the extraction and perfusion fixation are critical for tissue preservation. When any of these stages fails, the resulting samples (as the one shown) present partially collapsed regions that cannot be recovered by the pressure system.

The bronchiolar ducts are maintained in all the cases. The conductive airways are unobstructed, with the expected diameter for the bronchus divisions (~1 cm). The width of the alveolar walls also measures within the typical range reported by literature^37^. In Fig. 3a,b,d the oesophagus is visible in the centre of the sections (orange dashed circles). In the histological cuts of infected mice (Fig. 3c,d), we observe patchy areas of alveoli filled with inflammatory cells (L red letter). The destruction of lung tissue is also visible, along with diffuse oedema associated with the accumulation of a larger number of inflammatory cells.

The whole organ presents contrast-agent uptake. Higher pool concentrations and background padding are found proximal to the tuberculosis lesions.

**Figure 4:**
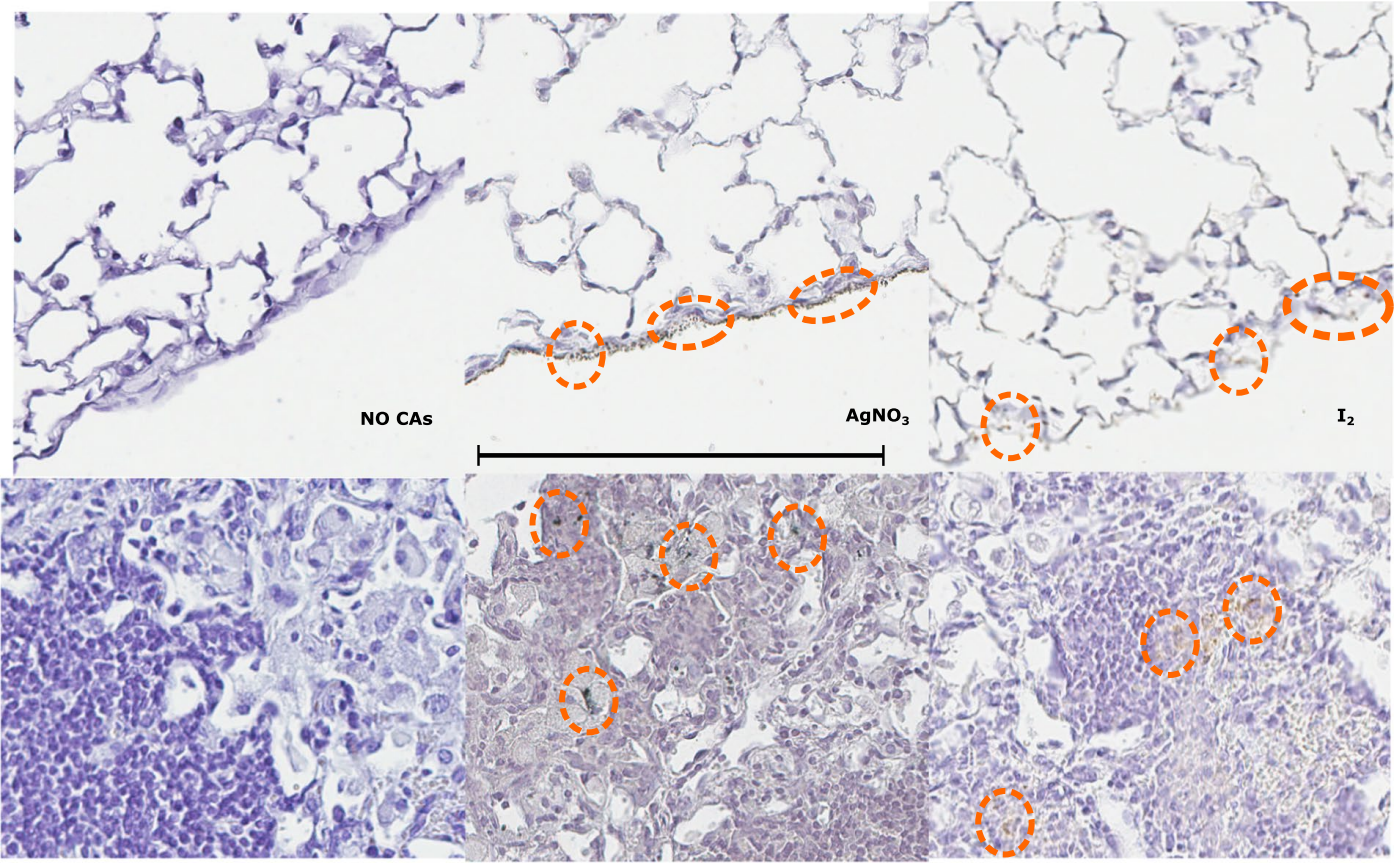
Presence, extent, and appearance of the contrast agent deposition in tissue areas of the histology presenting abnormal density (i.e., blood vessel walls, airway walls, tuberculosis lesions) in the micro-CT images. All sections are stained with haematoxylin. Borders of the tissue (first row) and lesion regions (second row) are shown by columns for: No CA) no contrast agent, AgNO_3_) silver contrast agent, and I_2_) iodinated contrast agent. Orange circles indicate the pools of the contrast agents. A difference on the background colour is also noticeable. The scale bar represents 200 um.

To obtain a detailed view of the contrast agent deposition, we acquired high-resolution histology images of tissue borders (first row, Fig. 4) and tuberculosis lesions (second row, Fig. 4). All sections are stained with haematoxylin (left column: no contrast agent; central column: silver contrast-agent; right column: iodine contrast agent). The lung cells remain intact both for healthy and diseased tissue. The lungs with silver nitrate are black, and the lungs with iodine are yellow on visual inspection. Neither of the contrast agents interferes with the histological counterstain. The contrast agent presence is visible as scattered pools distributed extensively on every tissue (encircled in orange in Fig. 4), which corresponds with the overall attenuation increase of the whole organ in the x-ray based virtual slicing. The deposition on the edges is responsible for the intensity frontier between the embedding media and the organ in the micro-CT scan. Silver nitrate and iodine are ionic contrast agents that promote the precipitation of proteins and chlorides^38,39^. Thus, significantly higher pool concentrations and background padding are found proximal to clusters of cells, targeting the structures with the abnormal densities (i.e., tuberculosis lesions) identified in the corresponding micro-CT slices. While these findings are not specific for tuberculous lesions, the trend towards adhering to highly populated regions is consistent with the contrast enhancement in the CT scans associated with inflammatory tuberculosis lesions which are already visible at week 8 after inoculation^37,40^.

The hollow tissue decreases in volume when embedded in ethanol and further decreases when embedded in paraffin. However, the volume of the lesion core remains consistent.

**Figure 5:**
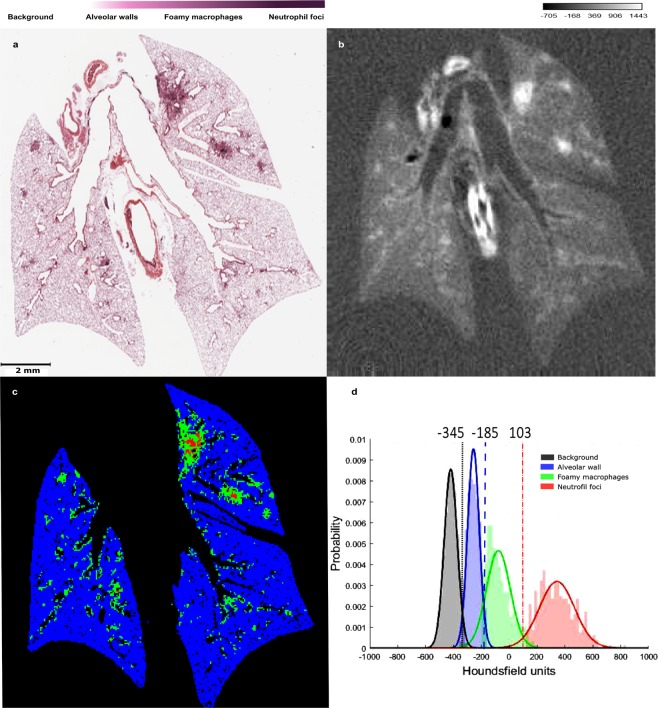
Illustration of the automatic segmentation procedure for the micro-CT scan of an infected lung with iodine uptake. The sample thumbnail of the histological slide (**a**) is registered with the micro-CT slice (**b**). The corresponding segmentation (**c**) was acquired by statistically modelling the HU distribution and translating the histopathological segmentation to micro-CT segmentation. The estimation of the thresholds of the granuloma cellular structure is shown by the grey level histogram. (**d**) The colour code for the intensity differences correspond with the lesion composition: the healthy parenchyma is in blue, the rim composed by foamy macrophages in green and the neutrophil foci in red.

The volume preservation of the relevant tissues (i.e., healthy lung, disease associated (lesion peripheral rim and central foci)) of the infected contrast-enhanced samples was estimated from the *in vivo* micro-CT scans and from those acquired on the excised organs embedded in ethanol and in paraffin. Figure 5 shows a sample thumbnail of the histological slide of the paraffin block (a) registered with the micro-CT slice (b) and the corresponding segmentation (c) for an infected lung with iodine uptake. Figure 5c is the automatic segmentation with the thresholds estimated from the HU histogram (d) and colour code: the organ is delimited with blue; in the lesions, the rim of macrophages is delimited in green and the neutrophil foci in red. The applied thresholds are specified in the HU histogram (d).

Our estimations show that the healthy lung tissue volume decreases slightly (14,3%) after the contrast-enhancing and embedding in ethanol, while the total diseased volume (including the peripheral rim and the central foci) decreases abruptly (53,7%). The former is due to the tissue dehydration and the latter is caused by the accurate delimitation of the peripheral rim edge enabled by the improved contrast. The hollow tissue (healthy parenchyma (31,8%), total diseased tissue (33,5%) and peripheral lesion rim (27,4%)) further decrease their volume after embedding in paraffin, as expected, while the lesion core formed by the infiltrated nuclei maintains its volume. For a graphical representation of the estimated volumes we refer the reader to Supplementary Fig. 3.

In the micro-CT scans of the contrast-enhanced samples, the largely increased width of the right tail of the intensity histograms and the corresponding growth of the edge contrast measure points to an effective contrast enhancement of the relevant lung structures and TB-related manifestations.

Both histological images and micro-CT images can differentially assess contrast agent uptake into normal parenchyma and into tuberculosis-infected lung sites in *postmortem* whole lungs. The increase in contrast of the *postmortem* micro-CT scans with contrast agents provides detailed images of the relevant lung structures (i.e., airways, blood vessels, and abnormalities). Those structures are unidentifiable in the *postmortem* micro-CT images of the control samples which were not enhanced with either contrast agent (Supplementary Fig. 4). For that reason, all subsequent metric comparisons refer to the *in vivo* micro-CT scans acquired before euthanizing the mice.

**Figure 6:**
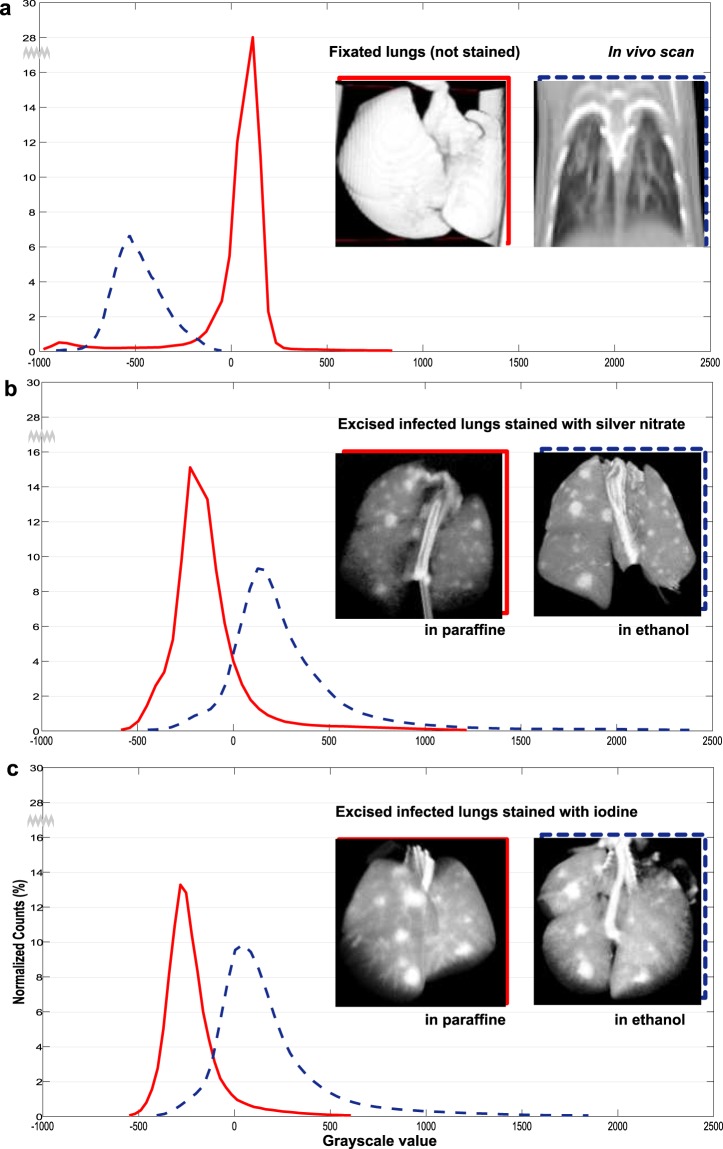
Quantitative measure of contrast estimated from the histogram of the micro-CT lung volumes. The histograms show the probability distribution of the voxel greyscale values (linear scale [HU]) from the concatenation of the segmented lung volumes available per condition. The axis legends are defined in the last graph and are the same for the three of them. A break in the y-axis is included from 16 to 28%. Renders of the micro-CT lung volumes from which the histograms have been computed are also shown The framing colour lines represent the legend of the graphs. Micro-CT scans of: (**a**) Lungs acquired *in vivo* (blue-dashed) and excised lungs without contrast agents (red); (**b**) Excised lungs with silver nitrate uptake acquired while embedded in ethanol (blue-dashed) and in paraffin (red); (**c**) Excised lungs with iodine uptake acquired while embedded in ethanol (blue-dashed) and in paraffin (red).

Figure 6 illustrates the contrast enhancement in the micro-CT images of the lung structures and tuberculosis lesions of the infected animals due to the contrast agent. Raw reconstructed tomographic volumes were used for analysis before any processing or re-slicing. Each histogram shows the probability distribution of the voxel greyscale values (linear scale [HU]) from the concatenation of the segmented lung volumes available per condition. The 3D reconstructions on the right are shown for illustrative purposes. From the histogram, the mean and standard deviation values of a Gaussian fit of the intensity probability distribution were computed (Table 2). With respect to *in vivo* scans, the histograms of lungs with silver nitrate uptake are shifted by 771 HU (354 HU) when embedded in ethanol (paraffin); the histograms of lungs with iodine uptake are shifted by 663 HU (261 HU) in ethanol (paraffin). This may be due to the relatively large voxel sizes of micro-CT imaging and the consequent partial volume effect which mixes the target tissues with the embedding medias (paraffin or ethanol). From our observations, the embedding material gets to fill the interalveolar septum and a pixel of size of 44 µm integrates multiple capillaries of the interalveolar septum. Paraffin presents a higher attenuation factor (mu) than ethanol as reflected in different shifts of the HU on the attenuation histograms. The abnormalities associated with the disease have intensities concentrated in the right tail of the histograms. The width of the right tail is measured as the difference between the 95^th^ percentile value and the mean (Table 2). The increase in the width of the right tail of the silver nitrate lungs (497.72% when embedded in ethanol and 322.41%, in paraffin) and of the iodine lungs (397.52% when embedded in ethanol and 198.61%, in paraffin) indicates that the contrast of the abnormal densities has improved. The differences between the embedding medias may be due to the partial volume effect and to the washing effect of the lungs being two hours submerged in hot paraffin before building the block.

**Table 2:** Metrics for the quantitative evaluation of contrast enhancement in lung tissue.

	Embedding	Histogram Gaussian fit (HU)	Right tail width (HU)	ECC increase (%)
*In vivo*		μ = −481, σ = 134	394.9	—
Excised not enhanced		μ = 56, σ = 241	711.2	—
Excised with silver nitrate uptake	in ethanol	μ = −127, σ = 252	1965.5	71.8
	in paraffin	μ = 290, σ = 391	1273.2	33.1
Excised with iodine uptake	in ethanol	µ = −222, σ = 155	1569.8	62.85
	in paraffin	µ = 182, σ = 323	784.3	25.66

Gaussian fit of the intensity histograms, width of the right tail of the histograms and the increase in edge contrast (ECC) of the micro-CT lung volumes available per condition (see Fig. 6).

Finally, the Edge Contrast Criterion (ECC) is computed on the four infected samples. For each embedding and each contrast agent, we considered the mean of the measurements for 31 slices per micro-CT volume of a lung region of interest with size 3.6 × 5.5 mm^2^. The ECC metric reveals contrast enhancement at relevant lung structures (Table 2).

## Discussion

We have demonstrated that the contrast-agents are able to increase the attenuation of the lung lesions caused by tuberculosis in x-ray based virtual slicing. The acquisition of *postmortem* whole lungs allow the 3D assessment of the characteristics of abnormal bronchoalveolar tissue. We showed that this approach is not limited to a single contrast agent by presenting comparable results with silver nitrate and iodine solutions. In addition, we demonstrated that our contrast-enhancing technique is compatible with disinfection protocols to inactivate pathogenic bacteria and allows subsequent analysis using classical histology. Our protocol does not introduce any additional shrinkage of the tissue other than that expected from the chemical drying procedure and paraffin embedding typical of standard histological examinations.

Regarding the imaging platform, we found that the advantage of micro-CT over 3D high-resolution microscopy imaging techniques is that the organs do not have to undergo the clearing process step required with single plane illumination microscopy^41^. Additionally, using micro-CT imaging for 3D virtual slicing avoids the complexity of working with 2D serial sectioning that requires the application of stereology to estimate alveolar volumes^31^ or the building of 3D reconstructions to perform histological analysis of the whole organ. As we also intend to use the contrast-agents in *in vivo* imaging and to achieve comparable results *in vivo* and *postmortem*, the micro-CT scan is the appropriate platform as it has enough resolution to visualize lung structures and large fields of view to fit murine models^42^.

It must also be considered that there is not an exact correspondence between the histology image and a slice in the micro-CT. The voxel size of our 2D micro-CT slice is 44 um, meaning that the image is a flat cross-section which integrates all the structures within 44 um depth, whereas the width of the histology samples is 3 um. At the time of the experiment, the optimal trade-off between computational burden and resolution was reached at the voxel resolution of 44 um, which gave the best virtual slices resolution-wise. Aiming to achieve virtual histology in future studies, we will increase the computational capacity of our working station in order to achieve the required resolution for the excised stained lungs without binning.

The limitations intrinsic to the imaging platform available for this research, could also be overcome by alternative CT systems. Phase-contrast or dual-energy CT can increase the contrast-to-noise ratio for samples without contrast agent. However, these techniques are usually only feasible with synchrotron light sources^43–48^, which are only available in 73 facilities worldwide, thus preventing their use in high-throughput studies. Cutting-edge spectral systems are expected to demonstrate superior image quality, even with larger voxel sizes than standard CT systems, provided that the attenuation of subjects under study is enhanced^30,49^, which could be achieved by contrast-enhancing preparations such as the one we proposed.

The extent of the contrast-agent in the pulmonary tissue suggested that the radiological enhancement by Iodine and Silver is non-specific for tuberculosis lesions. Thus, this technique could be applied to any organ with the pathological manifestations of infectious diseases. The proposed contrast-agents tend to adhere to highly cell populated regions. Both contrast agents demonstrate low protein binding, thus explaining the clear depiction of vessels, airways, connective tissue, and lesions. The uptake of silver nitrate by soft-tissue samples is susceptible of pitfalls and artefacts, mainly due to the sensitivity of the salt towards oxidation and the formation of a layer of metallic silver on the surface of the sample^38^. The accumulation of metallic silver on the sample surface and the dark colour of the lungs suggested that the immersion in the contrast agent was too long and excessive background formed. Reducing the time of the contrast-agent immersion to remove excess silver ion and other chemicals prior to further processing is required.

Due to the need for contrast-enhanced samples for material decomposition techniques, we chose to use a silver-based contrast agent for lung enhancement, since silver has a low absorption edge value that is detectable with any spectral scan. Silver-based contrast agents also presented higher contrast in conventional CT, thus facilitating the depiction of the rim and central regions of the lesions. The major advantage that encouraged us to develop this technique for iodine was its widespread use in *in vivo* imaging. Thus, our post-mortem segmentation of the lesions enhanced with iodine can be translated to *in vivo* scans using the injectable form of the contrast-agent.

Many laboratories are actively working toward the development of contrast-enhancing techniques to use in combination with micro-CT for animal organs imaging^6,11,50^. Preparation techniques for murine organs try to avoid tissue collapse and enhance radiodensity by vascular perfusion with fixatives^6,51^ or formaldehyde inflation^50^. Our contribution in this line is the innovative computerized pneumatic circuit which overcomes the challenges of homogeneous uptake and tissue preservation even in the case of perforation.

The use of an automatic tissue processor that performs fixation, dehydration, and wax immersion treatment is widely applied in pathology laboratories. The contrast-enhancing step could be added to the cycle; however, the samples would be caged in the cassette, thus preventing the contrast agent from penetrating all lobes uniformly.

Comparison of lung volumes throughout the process shows the expected shrinkage reported in the literature^50,52,53^, with no additional shrinkage due to the contrast agents applied. We recommended that samples are stored in 70% ethanol to avoid further volume changes. An alternative to be considered is immersion in agarose gels, which do not wash out the contrast agent or dehydrate the sample^11^. In the case of paraffin storage, preservation can be assured and moreover, the lungs can be scanned with only a slight decrease in the image contrast while continuing to allow planning of the histological cuts to the regions of interest.

Silver nitrate produced higher contrast at a 3% concentration (saturated) than iodine at the same concentration on the analysed micro-CT images. A higher contrast could be achieved for iodine by increasing its concentration up to its saturation limit. Subsequent histopathology enabled us to perform a detailed CT examination of the lung anatomy and composition from the payload carried by the contrast materials^54^. Consequently, in the future, this method may enable stratification of abnormal CT densities (including ground-glass opacities) associated with a wide variety of pulmonary diseases in preclinical research. Any other model of injury, infection or tumorigenesis which also present clusters of cells as hallmarks of the host response could be enhanced by high-atomic-number contrast agents in micro-CT imaging.

Potential adverse structural or health effects of contrast media have been extensively studied over the last 20 years. As a result, the design of small-molecule image contrast agents has been highly optimized with successful examples such as lipid emulsions (Exitron 12000, Miltenyi Biotec) and nanoparticles (Mvivo Au, MediLumine). The physical, chemical, and pharmacological properties of contrast agents can be manipulated and modified to fit the desired application and its design specifications^8^.

In conclusion, the results obtained through our contrast-enhancement technique allows the discrimination of micrometre length complex lung structures for the stratification of tuberculosis lesions in mice samples. Probably the biggest advantage of this technique is the fast data acquisition and the non-destructive virtual slicing. Our method provides volumetric images by conventional preclinical CT scan, which is an imaging technique accessible to the general scientific community. We challenge previous procedures in overcoming the ground glass opacities of the pathological condition of our samples by diffusing the contrast agent into the Tuberculosis lesions. While here our technique has been demonstrated with *post mortem* imaging, it provides the basis for designing a low-osmolar contrast agent/saline that could be used for *in vivo* imaging of the lung.

## Supplementary information

Supplementary Material

## Data Availability

The datasets used and/or analysed during the current study are available from the corresponding author on reasonable request.
