# Supplementary material for: X-ray-based virtual slicing of TB-infected lungs
Source: Sci Rep. 2019 Dec 18;9:19404. doi: 10.1038/s41598-019-55986-y (PMC6920455; doi:10.1038/s41598-019-55986-y)
Supplement: Supplementary file 1 — Supplementary Material [file 41598_2019_55986_MOESM1_ESM.pdf]

X-ray-based virtual slicing of TB-infected lungs

Ana Ortega-Gil[1,2,3] , Juan José Vaquero[1,2] , Mario Gonzalez-Arjona[2] ,Joaquín Rullas[3] , Arrate Muñoz Barrutia[1,2]

1 Departamento de Bioingeniería e Ingeniería Aeroespacial, Universidad Carlos III de Madrid, Spain

2 Instituto de Investigación Sanitaria Gregorio Marañón (IiSGM), Madrid, Spain

3 Diseases of the Developing World, Infectious Diseases-Centre for Excellence in Drug Discovery (ID CEDD), GlaxoSmithKline, Madrid, Spain

Supplementary material

Supplementary Appendix A Computerized pneumatic circuit.

The samples remained inflated during the staining protocol at a constant pressure by means of the modified manometer shown in Supplementary Fig. 1. A simple and accurate method to measure low air pressure is to balance a column of liquid of known weight against it and measure the height of the liquid. This manometer has no moving parts and requires no calibration.

A microcontroller reads a calibrated differential pressure sensor, which is connected to the biological sample and controls the air flow with a pneumatic valve. When pressure in the samples surpasses the selected pressure by 0.1 cm/H_2O (20 cm/H_20 in our case), the valve closes. Thus, the pressure decreases in a controlled manner thanks to a flow valve. If the pressure falls 0.1 cm/H_2O below the desired value, the valve opens, allowing the air of the pump to reach the sample with controlled flow. The modified manometer has two different safety systems for preventing damage to the sample: a) the microcontroller, which has a watchdog timer that closes the valve in the case of a delayed response of the pressure sensor and resets the control loop; and b) an overpressure valve in the main duct, which ensures that the pressure will always be below the water level of the exhaust chamber.

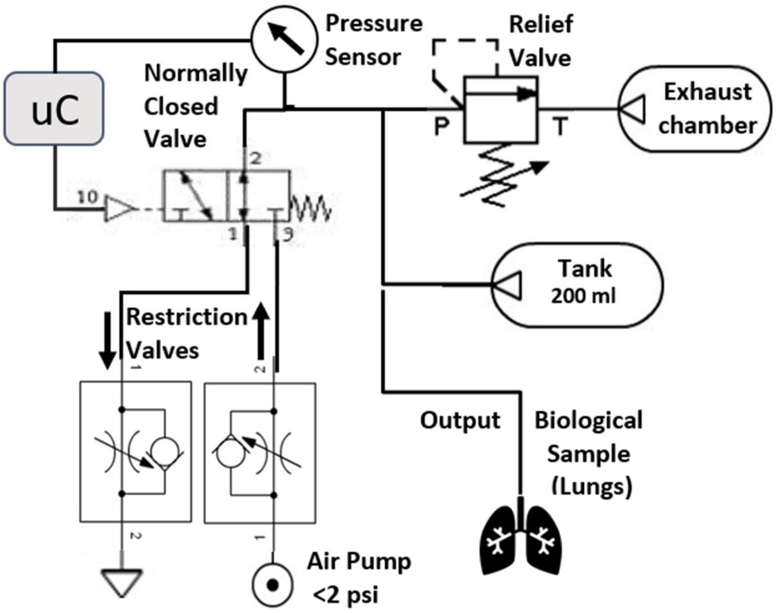

Supplementary Figure 1. Schematic of the pneumatic pressure system. The modified analogue manometer and the suspended lungs are connected through a catheter to a constant pressure of 20 cmH_2O. The microcontroller (uC) reads the analogue pressure sensor and manages the aperture of the normally closed valve using a hysteresis controller, thus limiting the pressure changes to 1 mmH_2O.

A. Graded Ethanol Concentration Fixation (GECF)

1. Create solutions of 50%,70%, 80%, 90%, 95%, and 100% Ethanol in different beakers.
2. Connect the computerized pneumatic circuit to the tracheal catheter in the lungs to the tube as shown in figure 1.
3. Submerge the lung, with the catheter now attached to the manometer into the beaker with 50% ethanol. Center the sample, without touching the borders.
4. Turn on the computerized pneumatic circuit and let the pressure equalize to 20 cmH2O constant pressure and keep the lungs inflated.
5. Leave the lungs in the solution for 1 hour.
6. After 1 hour has passed, switch the 50% beaker with the 70%. Keep the lung attached to the manometer. Dab the lungs onto a paper towel before switching every time.
7. After 1 hour, switch the beaker to the 80% ethanol one.
8. After 1 hour, switch the beaker to the 90% ethanol one.
9. After 1 hour, switch the beaker to the 96% ethanol one.
10. After 1 hour, switch the beaker to the 100% ethanol one.

B. Contrast Agent Immersion

1. Create solution of the contrast agent at 3% w/v, solved in ethanol.
2. After an hour of immersion in 100% ethanol, place the lungs (still attached to the computerized pneumatic circuit) into the beaker with the contrast agent (3%).
3. After 14 hours, wash them in 70% ethanol and store them in 70% ethanol.

Supplementary Table 1 Volume preservation

Supplementary Table 1 Threshold selection per stain for quantification of lung volumes. In vivo and ethanol-embedding thresholds manually selected to divide the grey level histogram into three parts. Paraffin thresholds automatically translated from the histology segmentation to the lesion compartments in micro-CT images. The lower range identifies the healthy tissue, the mid-range identifies the disease peripheral rim, and the upper range identifies the neutrophil foci.

	**In vivo**	**Ethanol**	**Paraffin**
Min	(Lung, Rim, Cup)	(Lung, Rim, Cup)	(Lung, Rim, Cup)
Silver nitrate stain	(-712, -417, -227)	(-100, 277, 1300,)	(-345, -198, 157)
Iodine stain	(-712, -417, -227)	(-100, 480, 1600)	(-345, -185, 103)

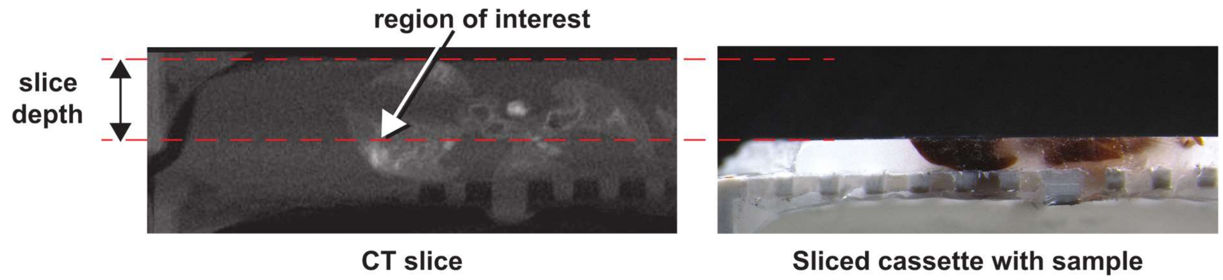

Supplementary Figure 2. Micro-CT scan of the paraffin block guides the histological slicing by providing the depth of the tuberculosis lesion from the paraffin surface. The sagittal view of the micro-CT scan (a) is used to measure the depth at which the microtome has to slice to reach the region of interest in the paraffin block (b).

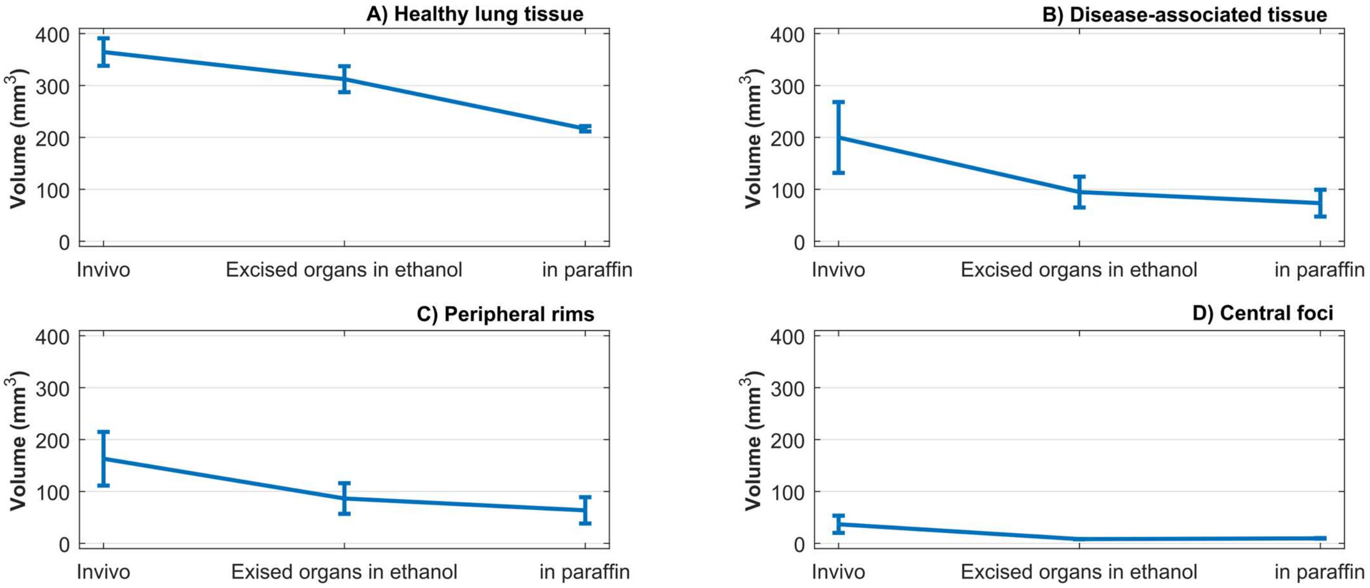

Supplementary Figure 3. Illustration of the lung volume preservation on the stained lungs (for both silver nitrate and iodine staining) embedded in ethanol and paraffin in comparison with those measured in the in vivo micro-CT scan: (a) Total healthy tissue volume of the lung (lower intensity regions in the micro-CT volumes discarding the airway tree); (b) Total diseased tissue including rims of macrophages and neutrophil foci (high intensity regions); (c) Detail of the peripheral rim volume (mid intensities); (d) Detail of the infiltration nuclei volume (maximum intensities). The difference in the in vivo diseased tissue volume with respect to the estimation on the stained lungs is due to the improved contrast: The peripheral rim edge contrast was enhanced with the metallic staining, giving a more accurate volume measurement.

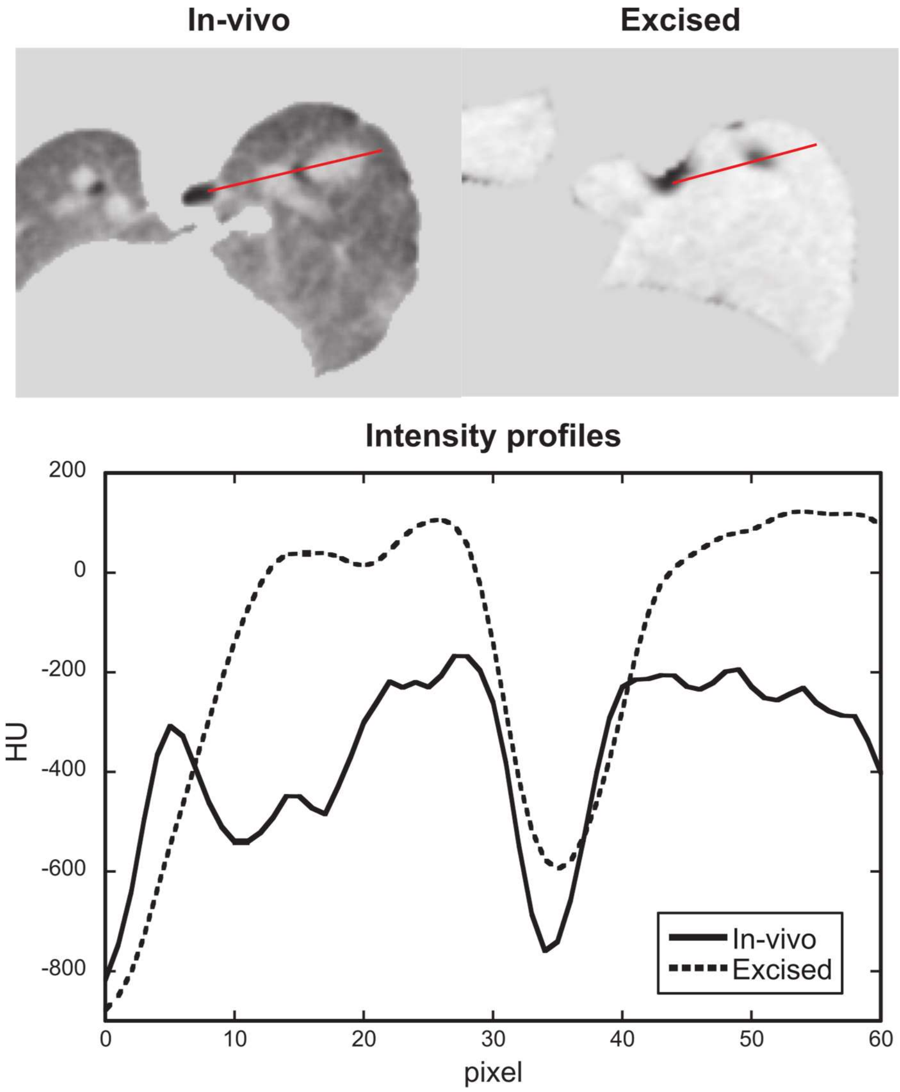

Supplementary Figure 4. Illustration of the low contrast and low structural information on micro-CT scans of excised lungs without metallic contrast agent staining. Example slice of a micro-CT lung scan acquired: (Left) in vivo (before euthanizing the mice) and (right) postmortem (without metallic contrast agent staining and embedded in ethanol). The intensity profiles (in HU) shown in the graph below corresponds to the red lines drawn on the micro-CT slices. The lowest intensities correspond to the absence of pulmonary tissue (background, airways). The variation of the highest intensities in the in vivo image match up with the pulmonary structures under study. The low contrast of the excised image prevents to appreciate those variations.
